# Supplementary material for: RAS at the Golgi antagonizes malignant transformation through PTPRκ-mediated inhibition of ERK activation
Source: Nat Commun. 2018 Sep 5;9:3595. doi: 10.1038/s41467-018-05941-8 (PMC6125387; doi:10.1038/s41467-018-05941-8)
Supplement: Supplementary file 1 — Supplementary Information [file 41467_2018_5941_MOESM1_ESM.pdf]

Supplementary Information

RAS at the Golgi antagonizes malignant transformation through PTPRk mediated inhibition of ERK activation.

Casar et al.

Supplementay Figure 1

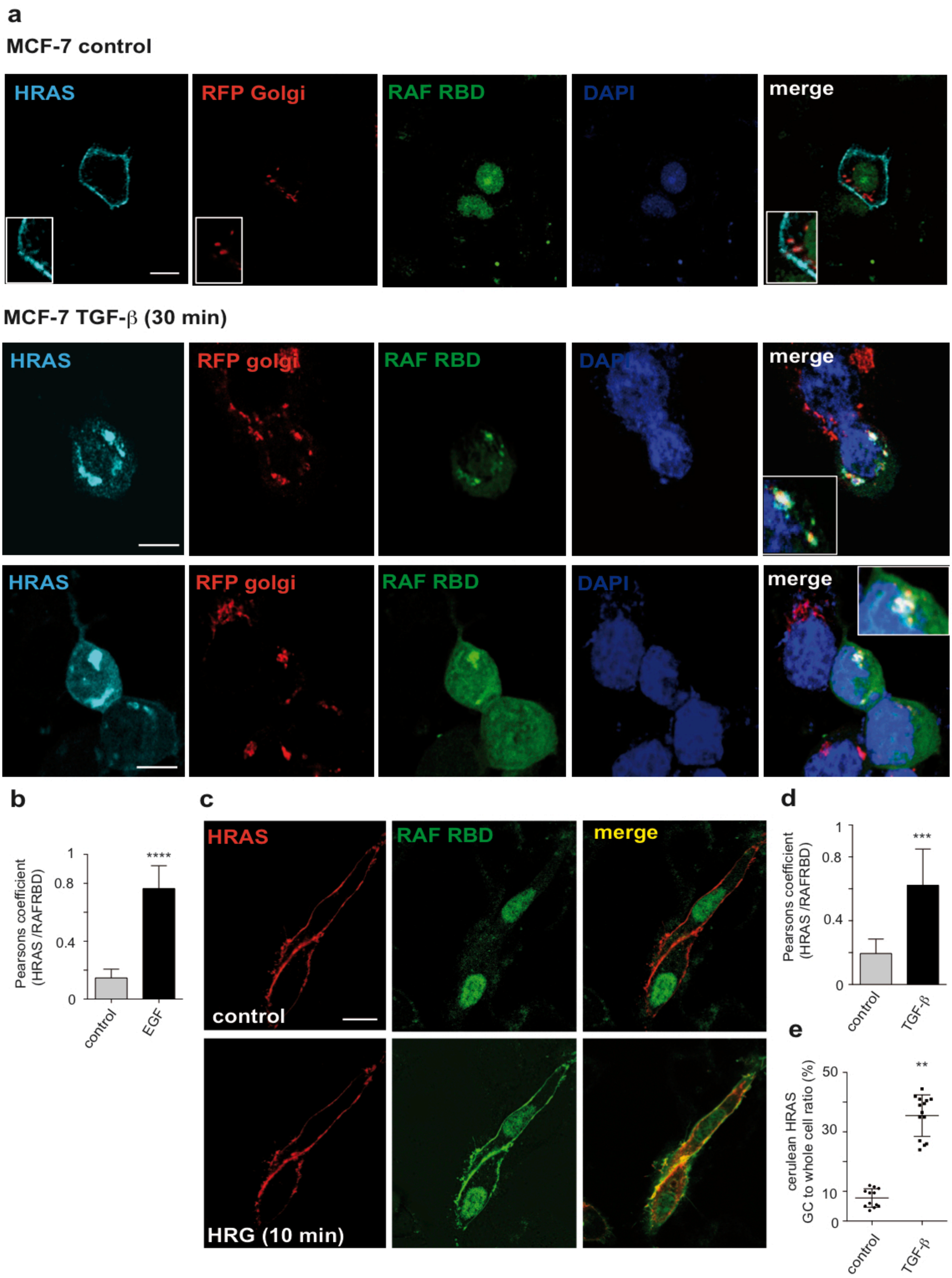

Supplementary Figure 1. RAS activation at the GC in response to apoptogenic stimulation. **a)** RAS activation in endomembranes in MCF-7 cells, control and treated with TGF-β. Cells were transfected with constructs expressing cerulean-HRAS and the RAS-GTP biosensor E3-R3 (RAF RBD) (1 μg each) and stimulated for 30 min. GC was revealed by the RFP Golgi probe. Insets show areas of prominent RAS-GTP accumulation in the GC. Scale bar = 10 μm. **b)** Pearson's correlation coefficient of mCherry HRAS and GFP RAFRBD colocalization at the plasma-membrane in control and EGF-treated cells (50ng/ml, 5 min). Data shows average ± SEM from 3 independent experiments, each with 12-14 fields/group and an average of 4-6 cells per random field. **** p <0.0001 by Student t-test. **c)** RAS activation by HRG is restricted to the plasma-membrane. MCF-7 cells transfected with constructs expressing cherry-HRAS and the RAS-GTP biosensor E3-R3 (RAF-RBD) (1 μg each) and stimulated for the indicated times. Scale bar = 10 μm. **d)** Pearson's correlation coefficient of cerulean HRAS and GFP RAF-RBD and RFP-Golgi colocalization in control and TGF-β -treated cells (5ng/ml, 30 min). Data shows average ± SEM from 3 independent experiments, each with 12-14 fields/group and an average of 4-6 cells per random field. *** p <0.005 by Student t-test. **e)** Cerulean HRAS activation at Golgi / whole cell ratio in control and TGF-β -treated cells. Data shows average ± SEM from 3 independent experiments, each with 12-14 fields/group and an average of 4-6 cells per random field. ** p <0.01 by Student t-test.

Supplementary Figure 2

a

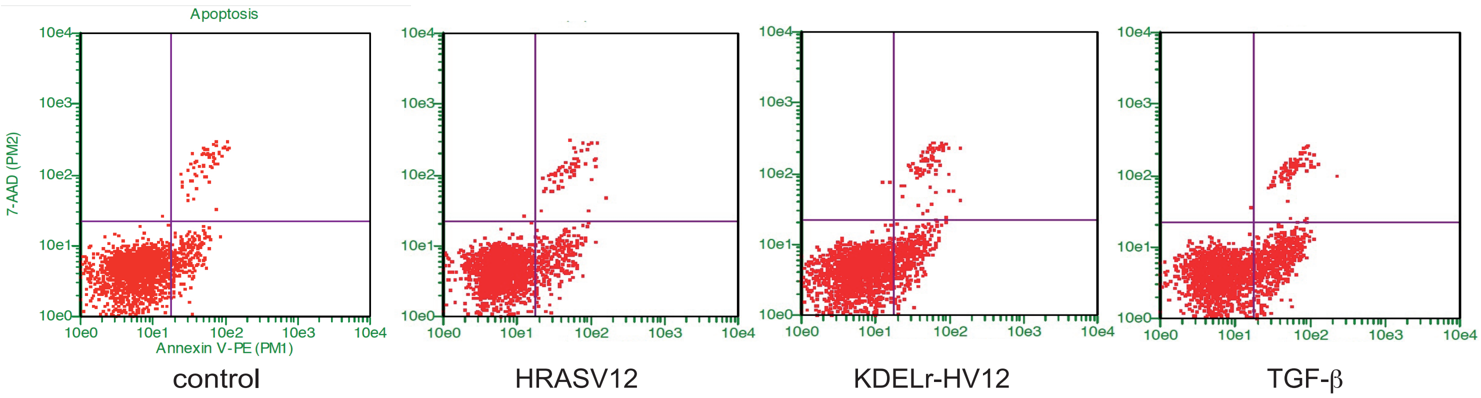

b

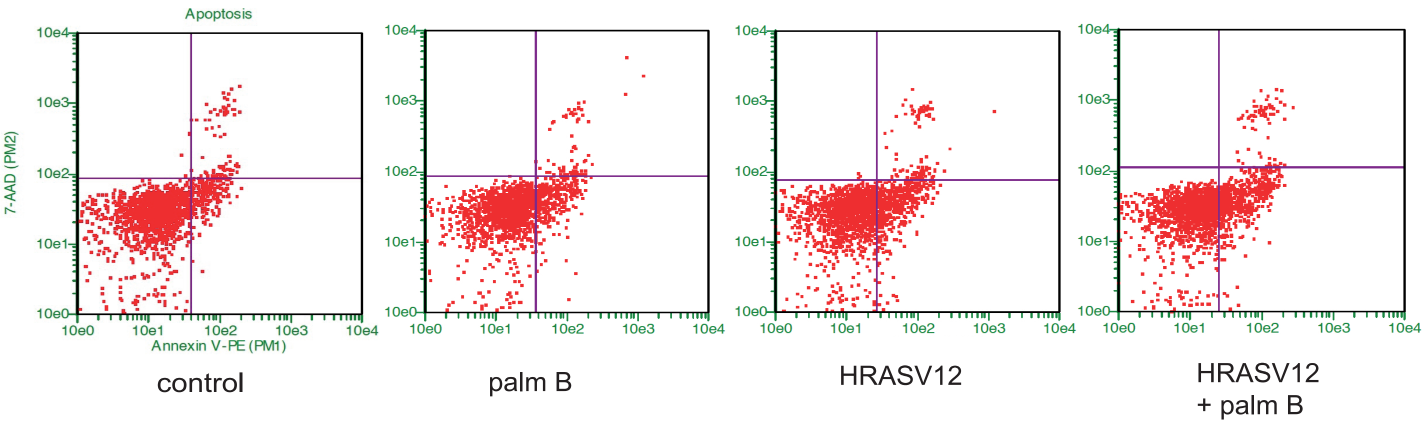

Supplementary Figure 2. Induction of apoptosis by RAS activation at the GC. a) Induction of apoptosis in MCF-7 cells transfected with the indicated constructs (1 μg each) or treated with TGF-β (5ng/ml for 12 hrs). **b)** Apoptosis in response to palmostatin B treatment (10 μM, 24 hrs) in cells transfected with the indicated constructs (1 μg each). In both cases apoptosis was evaluated by annexin V detection using the Guava /nexin assay.

Supplementary Figure 3

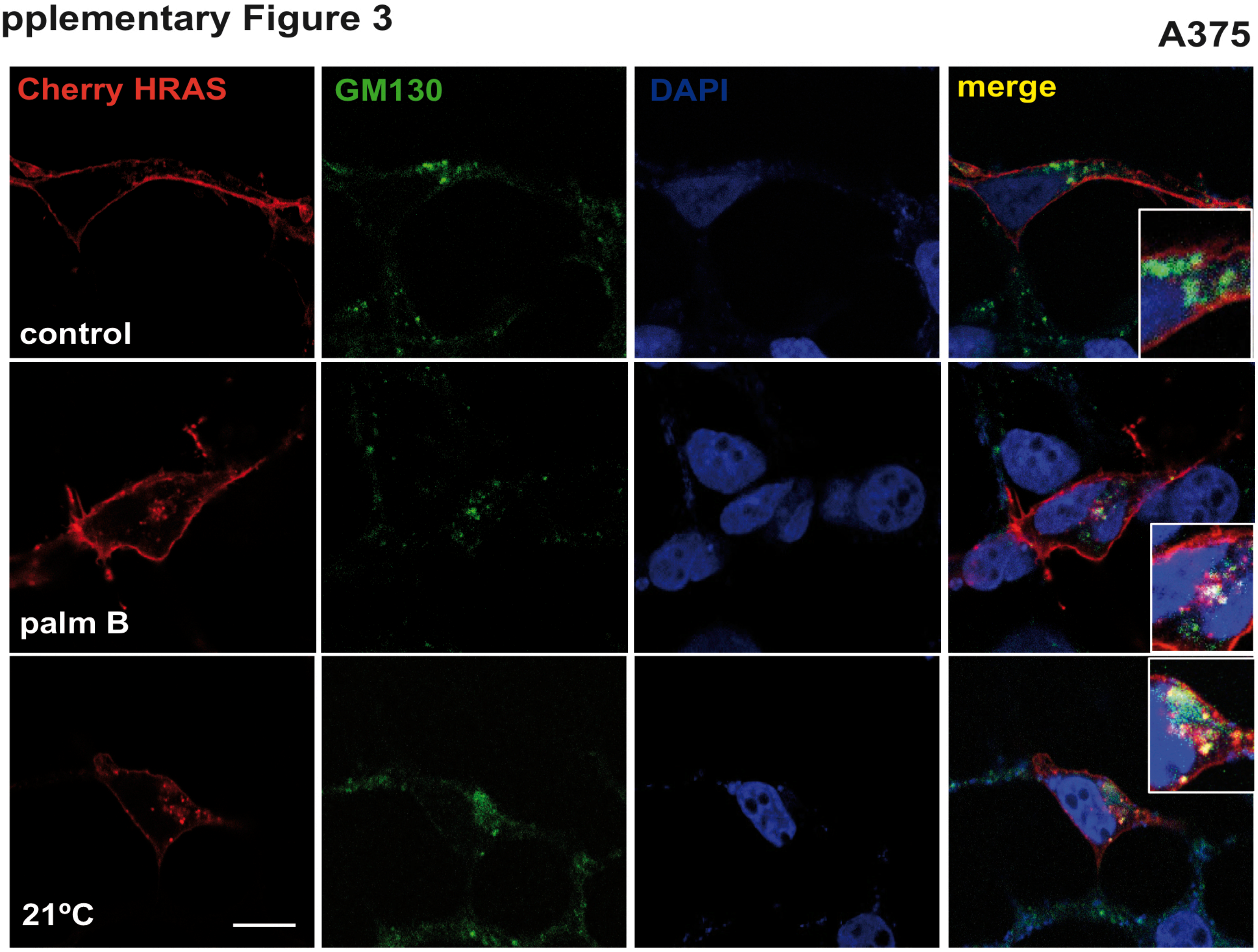

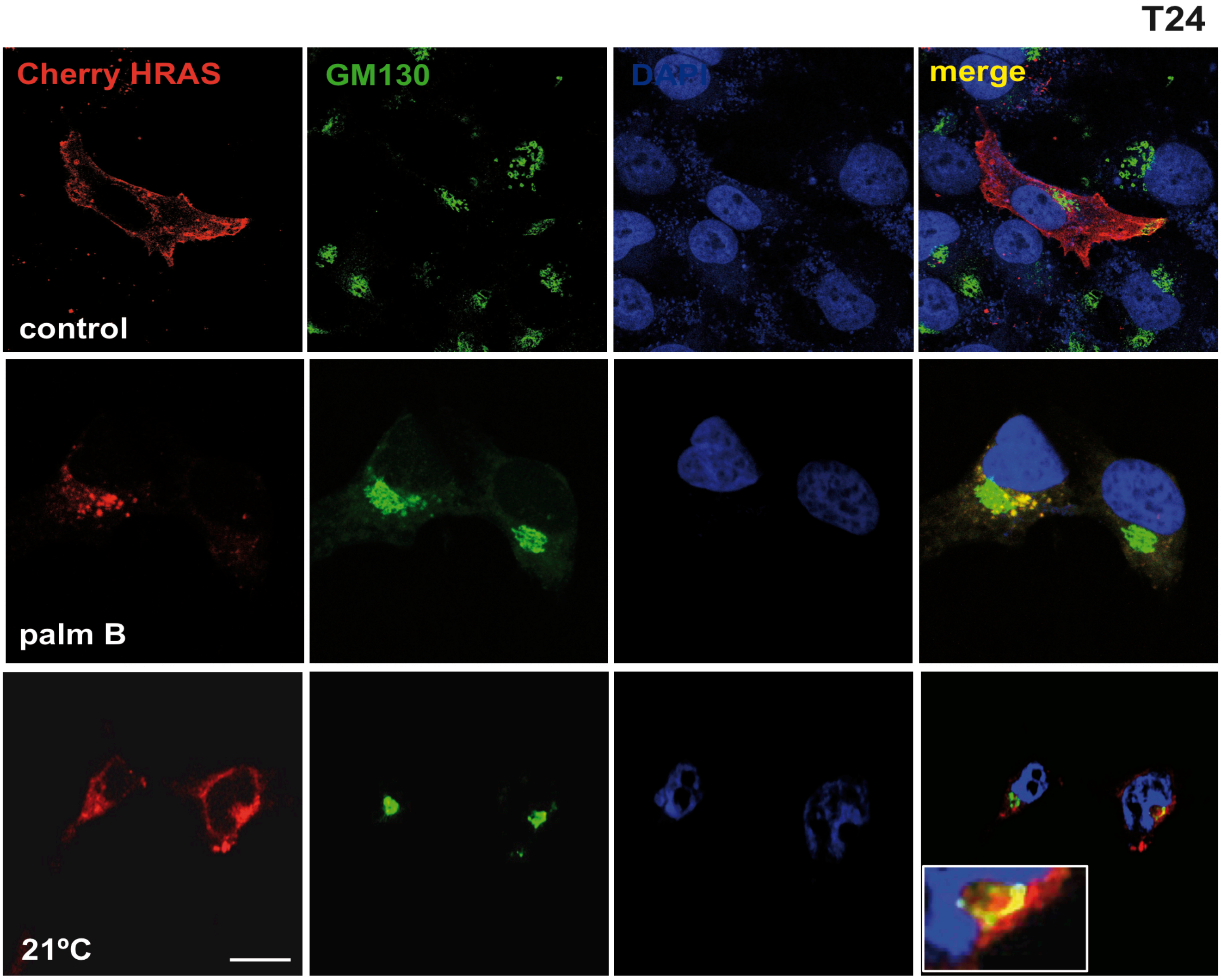

Supplementary Figure 3. RAS translocation to the GC in response to palmostatin B and 21ºC treatments. RAS sublocalization was analyzed in A375 and T24 cells, control and treated with palmostatin B (10 μM, 24 hrs) o cultured at 21ºC for 24 hrs. Cells were transfected with cherry-HRAS (1 μg). GC was revealed by GM 130 staining. Insets show areas of prominent RAS accumulation at the GC. Scale bar = 10 μm.

Supplementary Figure 4

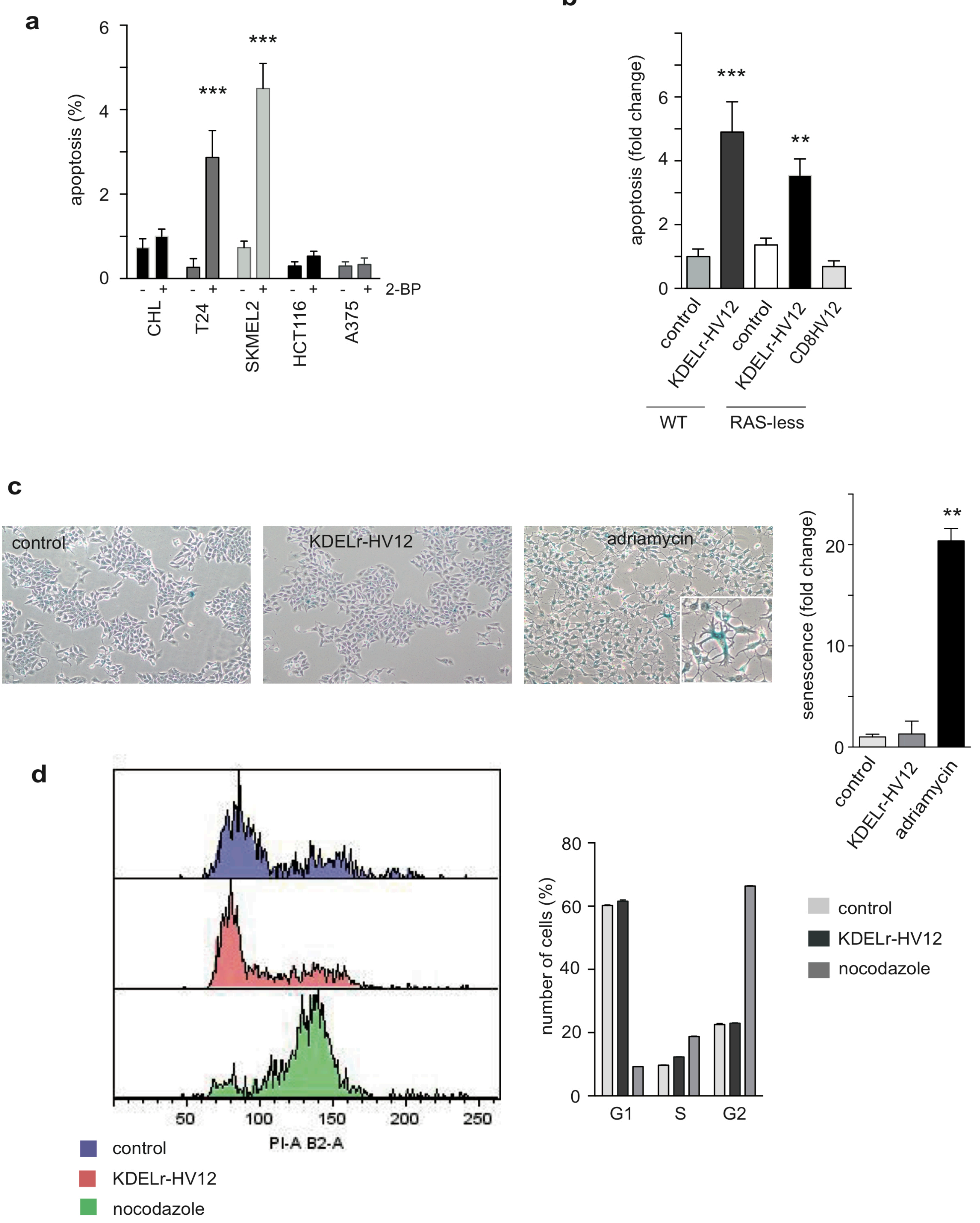

Supplementary Figure 4. Induction of apoptosis by RAS activation at the GC. A) Apoptotic response to 2-bromopalmitate (100μM, 24 hrs) of the indicated cell lines. **B)** Induction of apoptosis in wild type (WT) and Ras-less fibroblasts transfected with the indicated constructs (1 μg each). Ras-less fibroblast were treated with tamoxifen 600nM for 5 days to ablate KRas previous transfection. **C)** GC-RAS (1 μg) effects on senescence in MCF-7 cells. Adriamycin (1μM, 24 hrs) was used as a positive control. A-C data shows average ± SEM from 3 independent experiments. ** *p* <0.01; *** *p* <0.005 by Student t-test. **D)** GC RAS (1 μg) effects on cell cycle progression in MCF-7 cells. Data shows average ± SEM from 3 independent experiments. ** *p* <0.01; *** *p* <0.005 by Student t-test.

Supplementary Figure 5

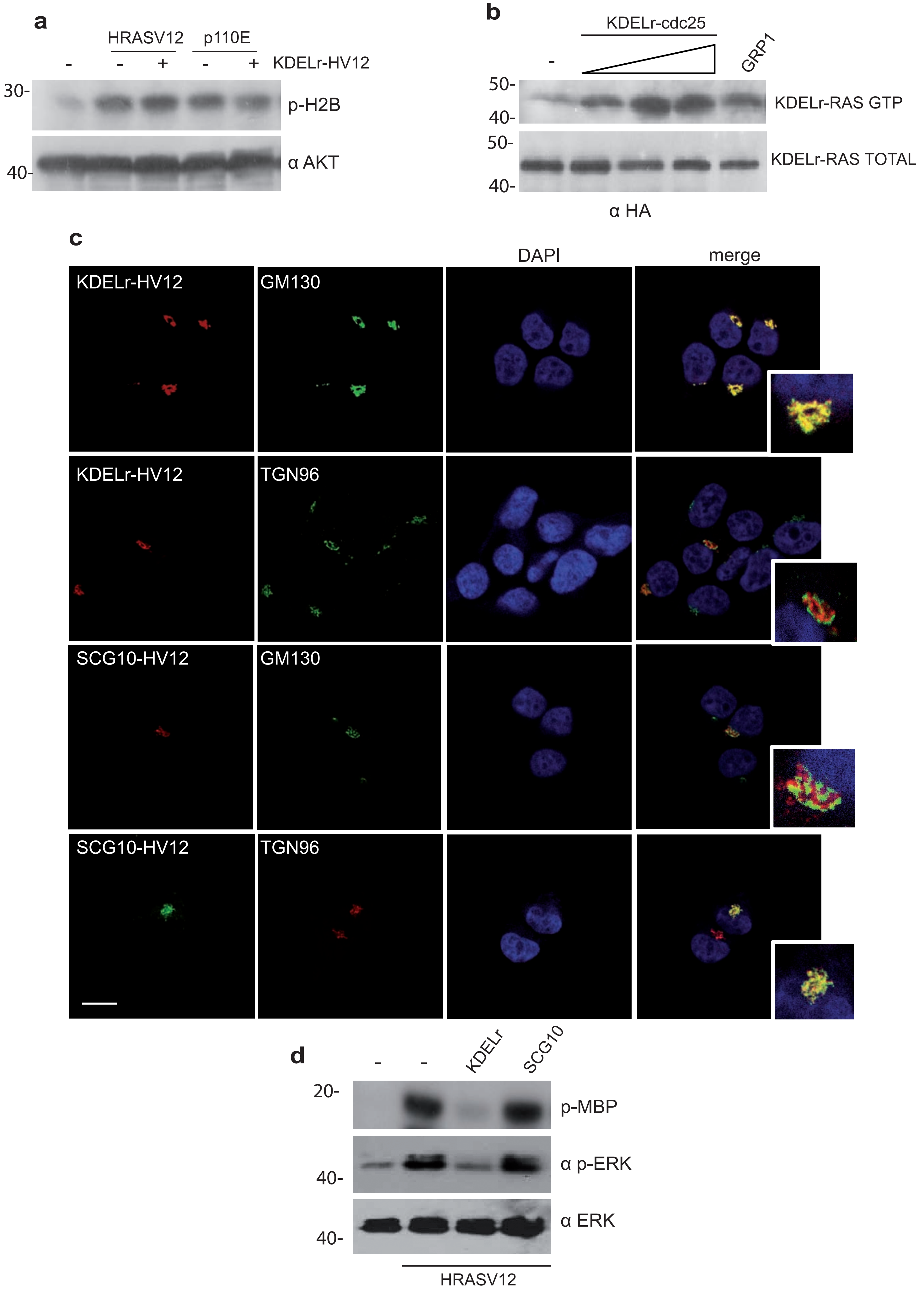

Supplementary Figure 5. Effects of RAS activation at the GC on ERK phosphorylation. A). AKT kinase activity levels in MCF-7 cells co-transfected with constitutively-activated HRAS or PI-3K (p110 subunit) (1 μg) with (+) or without (-) KDELr-HV12 (1 μg). AKT kinase activity was assayed using histone H2B as substrate. **B)** KDELr-CDC25 activates RAS at the GC. MCF-7 cells transfected with HA-tagged KDELr HRAS (0.5 μg), unstimulated (-) or transfected with increasing concentrations (0.25-1 μg) of KDELr-CDC25. Cells transfected with RASGRP1 (1 μg) serve as positive control. GTP loading was assayed by GST-RBD (RAF) pull-down (KDEL-RAS GTP). **C)** Localization to the cis- and trans-golgi of KDELr- and SCG10-HV12 respectively. Confocal images of MCF-7 cells transfected with the indicated constructs co-stained with GM130 or TGN96 as cis- and trans-GC markers respectively. Scale bar = 10 μm. **D)** Effect of trans-Golgi RAS signals on ERK phosphorylation and activity induced by HRASV12. in MCF-7 cells were transfected with the indicated constructs (1 μg). ERK phosphorylation was assayed by immunoblotting. ERK kinase activity was assayed using MBP as substrate.

Supplementary Figure 6

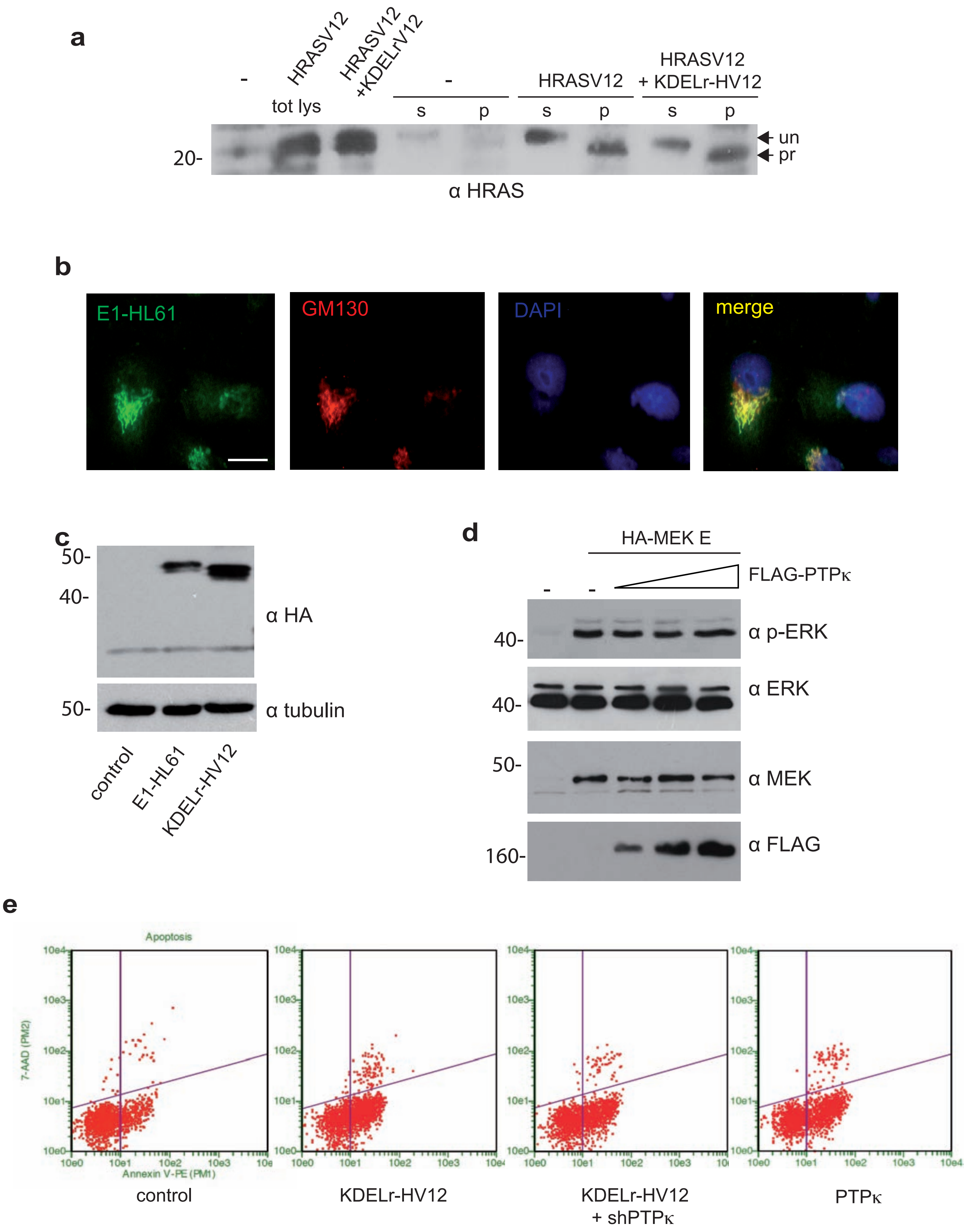

Supplementary Figure 6. GC RAS effects on ERK activation via PTPRk. A) Effect of GC RAS signals on RAS levels at the plasma-membrane. RAS levels at the soluble (S) and particulate (P) fractions of MCF-7 cells transfected with the indicated constructs. Arrows (un/pro) indicate processed and unprocessed RAS forms. **B)** E1-HL61 localization at the cis-Golgi in MCF-7 cells transfected with the construct (1 μg). GC was revealed by GM 130 staining. Scale bar = 10 μm. C) comparative expression levels of the Golgi-tethered constructs (1 μg) in MCF-7 cells. **D)** Effects of PTPRκ expression on ERK activation induced by MEK E. Cells were transfected with MEK E where shown, together with increasing concentrations (0.25-1 μg) of a construct expressing PTPRκ. **E)** Induction of apoptosis in MCF-7 cells transfected with the indicated constructs (1 μg each), evaluated by annexin V detection using the Guava /nexin assay.

Supplementary Figure 7

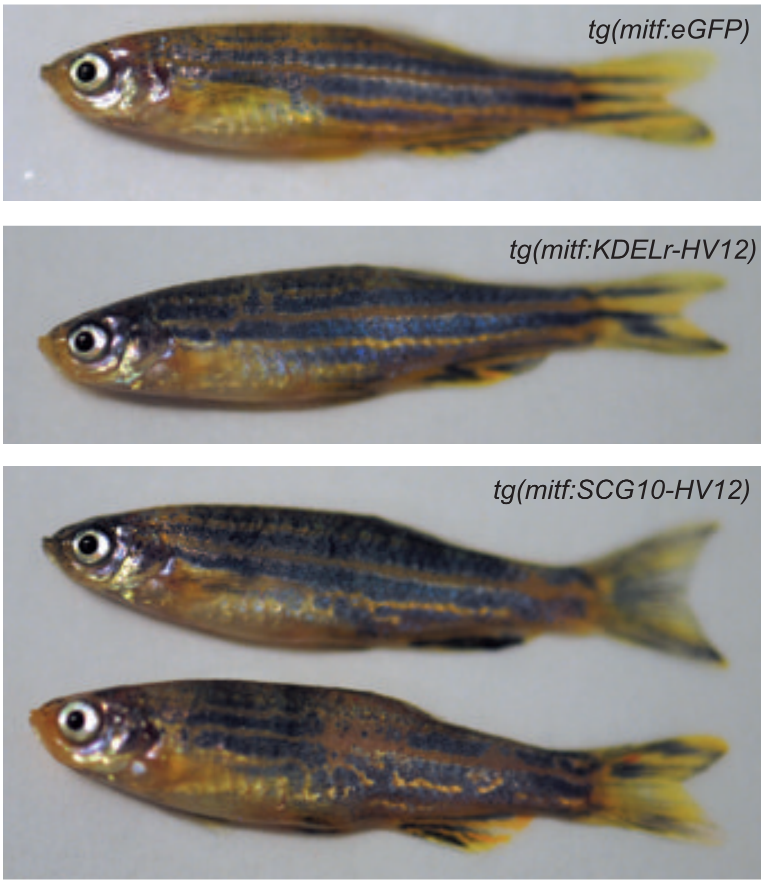

Supplementary Figure 7. Macroscopic view of representative fish expressing the indicated HRASV12 site-specific transgenes after 14 weeks.

Supplementary Figure 8

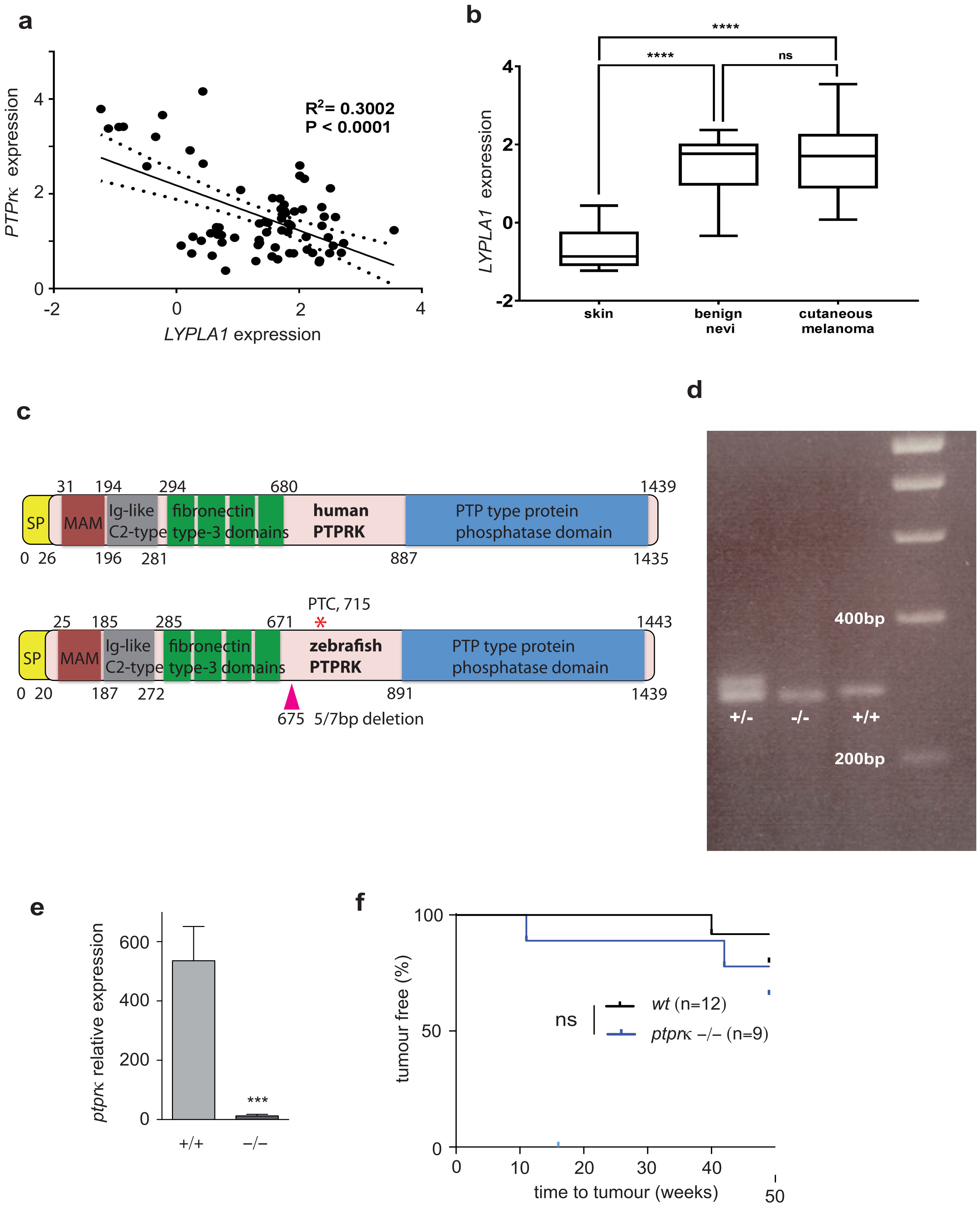

Supplementary Figure 8. PTPRk is a tumor suppressor in melanoma. A) Correlation of *PTPrκ* and LYPLA1 (APT-1) expression levels in normal skin (n=7), benign nevi (n=18) and cutaneous melanoma samples (n=45) generated using an available gene dataset, accessed through the Oncomine platform. **B)** Analysis of LYPLA1 (APT-1) expression in the samples from A. ***p <0.001 ****p <0.0001 by Student t-test. **C)** The CRISPR-Cas9 system was used to generate a 5 and 7 base pair deletion in the *ptprκ* locus from amino acid 675, predicted to encode a premature truncating codon after 714 amino acids. **D)** The 5bp and 7bp deletions resolved on a 3% agarose gel. **E)** qRT-PCR demonstrates *ptprκ* mRNA is significantly down-regulated in *ptprκ* mutant embryos 6 hours post fertilisation. **F)** Kaplan-Meier plot of protruding tumour incidence in *ptprκ* wild-type nacre animals (n=12) compared to *ptprk* -/- (n=9) expressing BRAFV600E specifically in melanocytes. ns - not significant by Mantel-Cox test.

Supplemental Figure 9

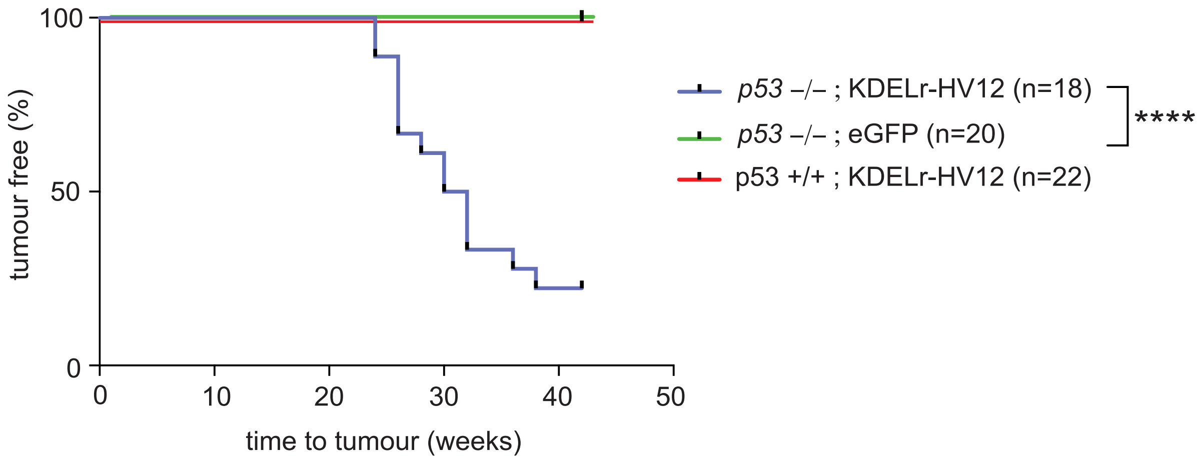

Supplementary Figure 9. tp53 status determines GC-RAS induced melanomagenesis. Kaplan-Meier plot of protruding tumor incidence in *p53* mutant nacre animals (n=20, green line) injected with EGFP or p53 wild-type nacre animals injected with KDEL-H12V (n=22, red line), compared to *p53* mutant nacre animals (n=18, blue line) injected with KDEL-H12V. ****p <0.0001 by Mantel-Cox test.

Supplementary Figure 10

Figure 1

a

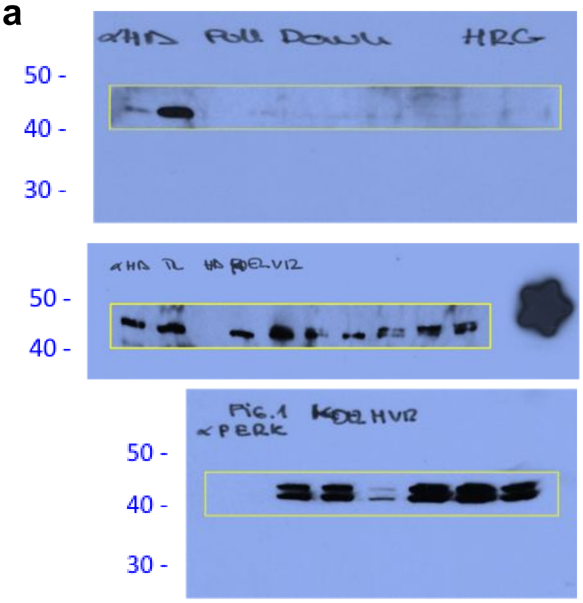

Figure 2

a

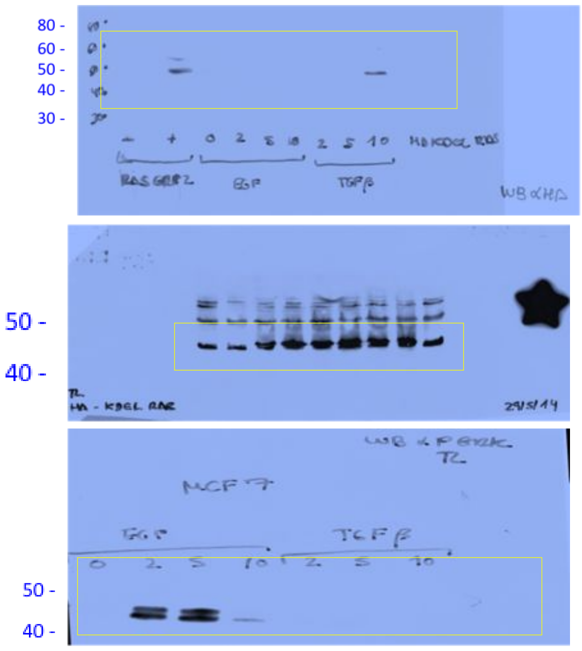

Figure 3

a

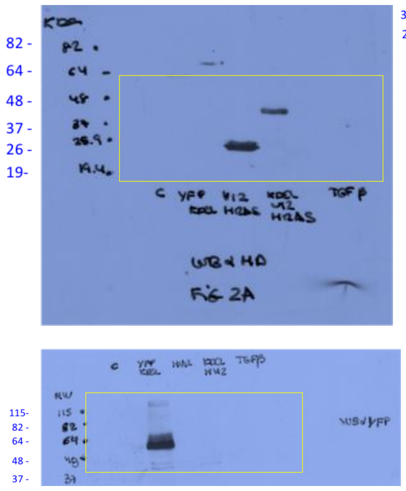

b

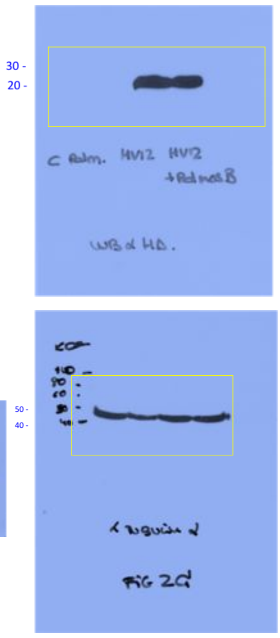

c

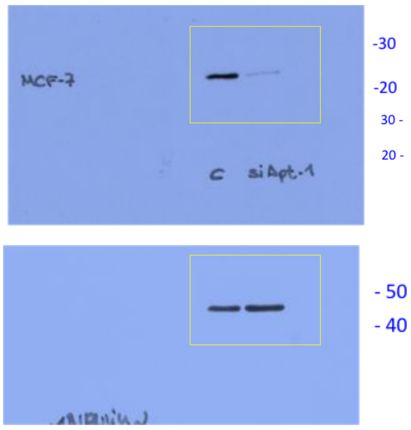

e

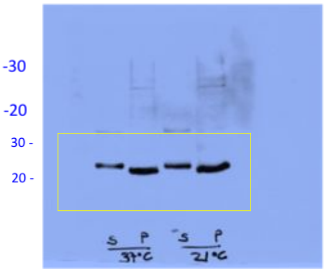

Figure 4

a

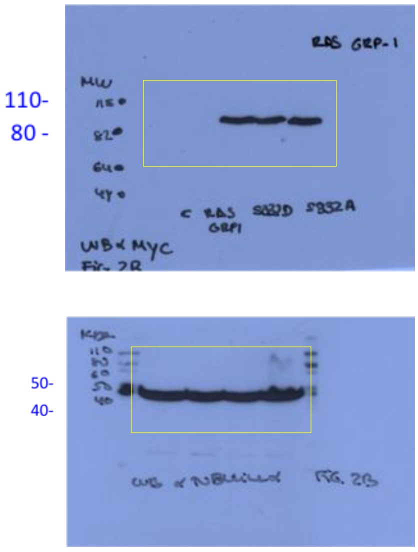

b

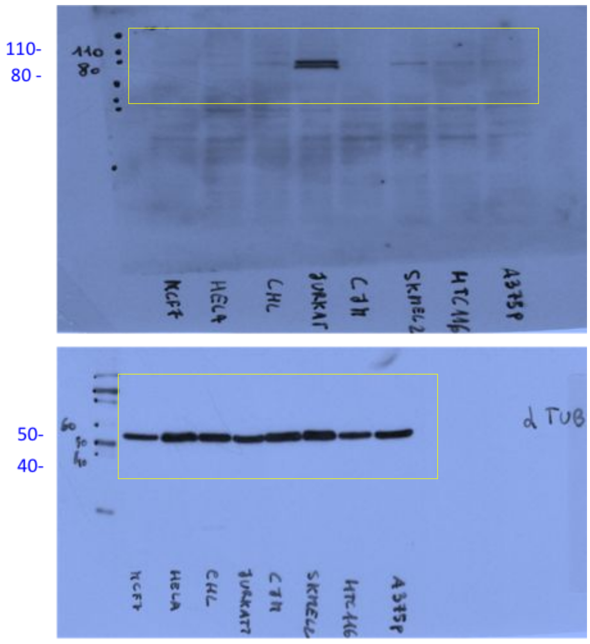

c

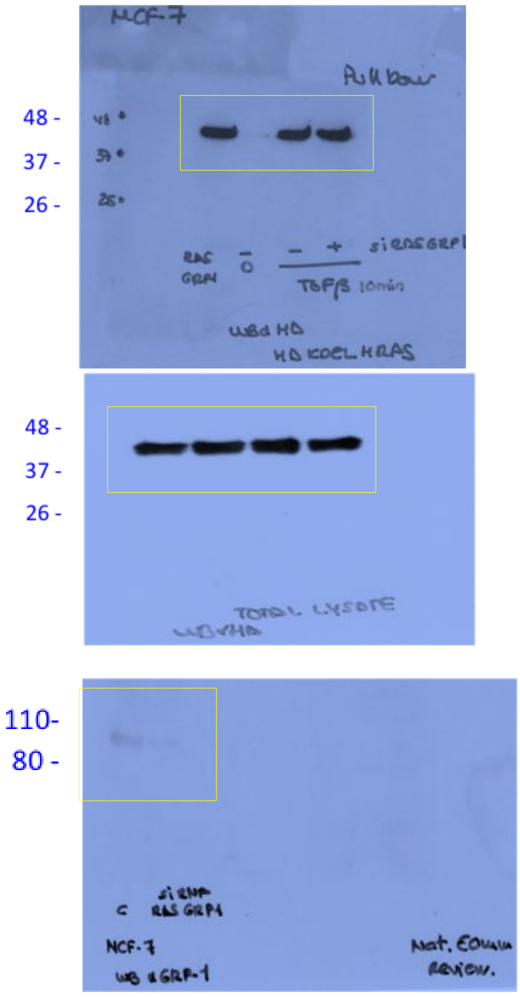

Figure 5

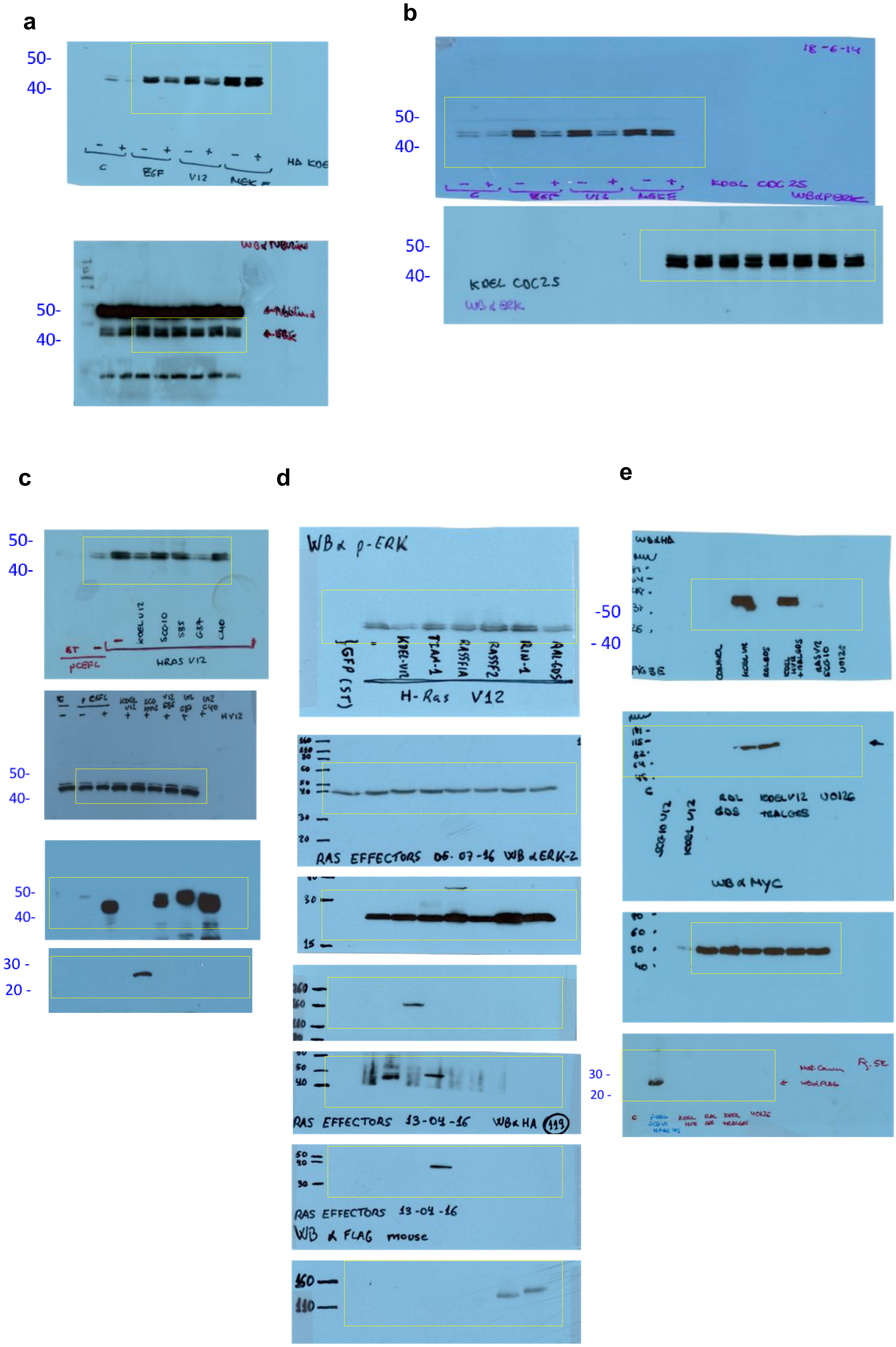
a
b
c
d
e
50-
40-
HA KDEL
C
EGF
V12
MEK
WB α TUBULIN
18-6-14
KDEL CDC25
WB α pERK
WB α ERK
HRAS V12
pCEFL
KDEL V12
SCO-10
S35
G37
C40
WB α p-ERK
H-Ras V12
GFP (ST)
TIAM-1
RASSF1A
RASSF2
RIN-1
RAL-GDS
RAS EFFECTORS 05-07-16 WB α ERK-2
RAS EFFECTORS 13-04-16 WB α HA
RAS EFFECTORS 13-04-16
WB α FLAG mouse
WB α HA
WB α MYC
-50
- 40
30 -
20 -
U0126

Figure 6

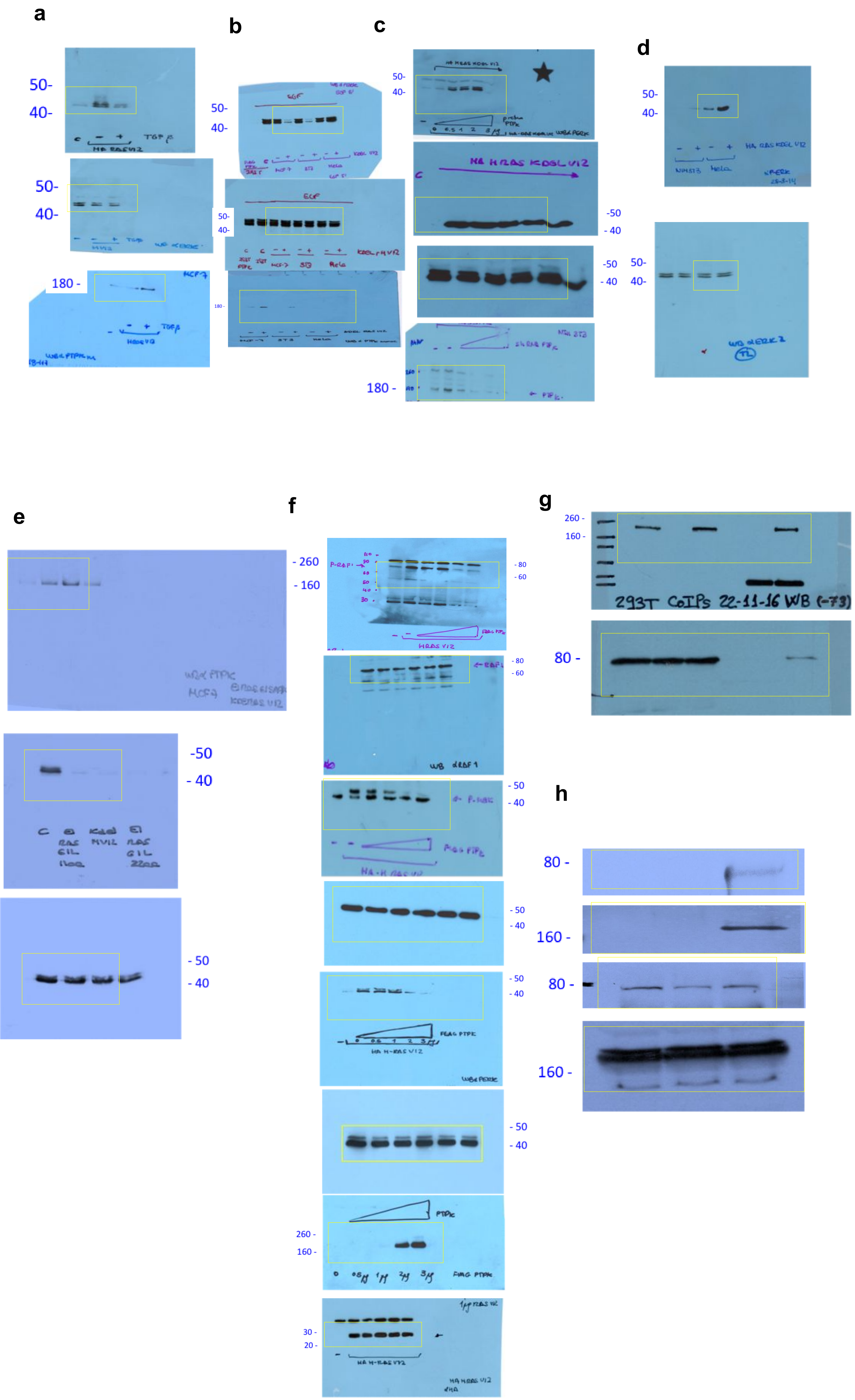

Figure 7

a

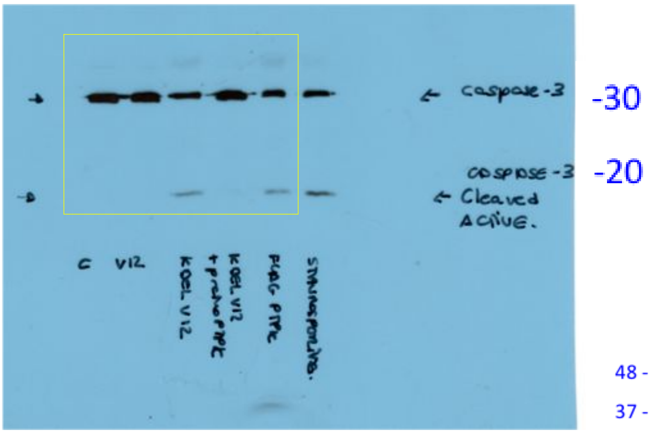

b

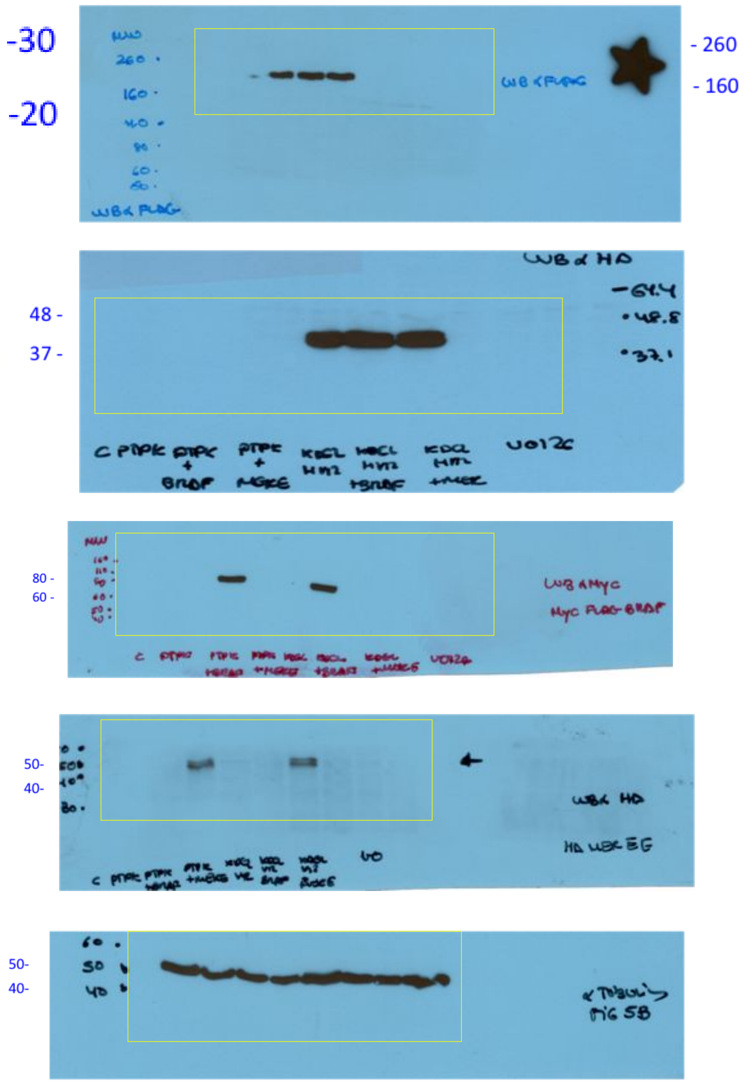

Figure 8

a

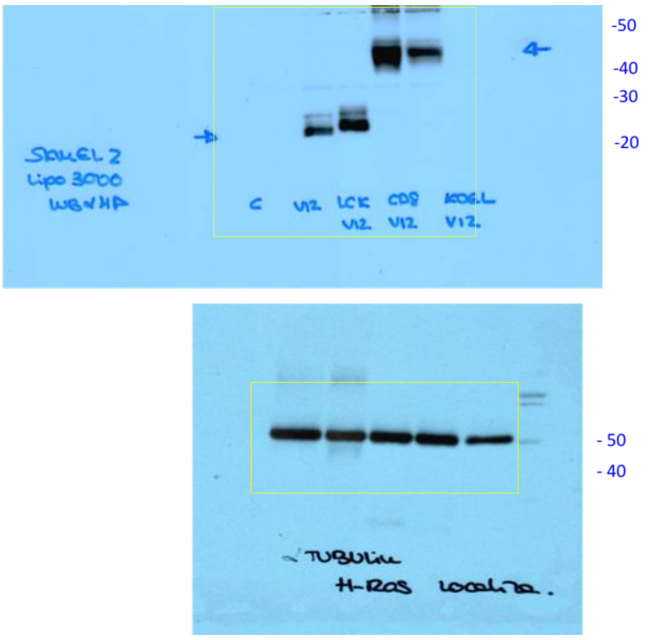

Figure 10

c

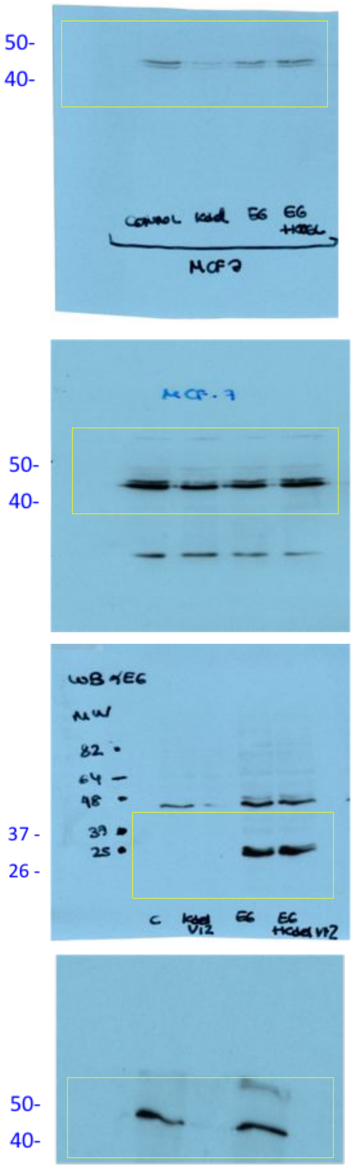

d

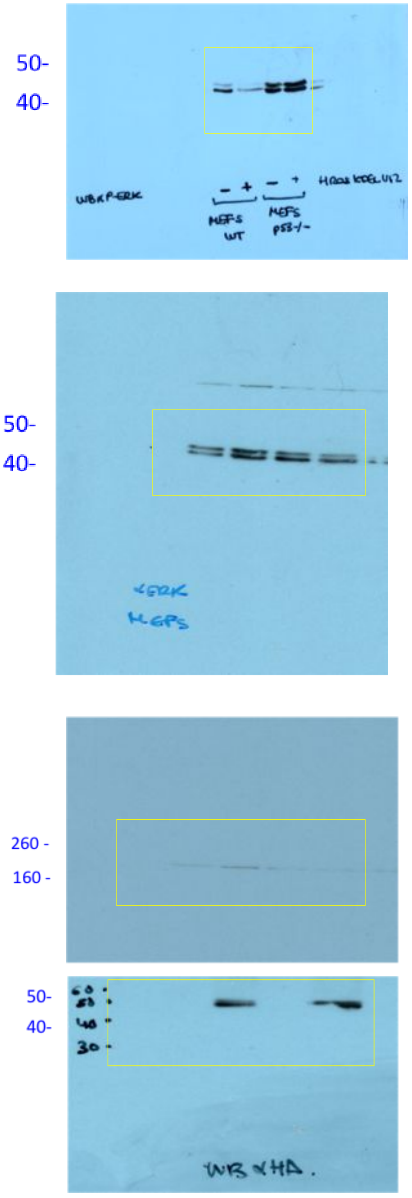

Supplementary Figure 10 .Uncropped original blots used in Figure 1, 2, 3, 4, 5,6,7,8,10

	vector	KDELrHV12	KDELr GFP	foci / μDNA
H-Ras V12	4860	1960(-40%)	4600 (-6%)	
M1 V12	9515	2920 (-70%)		
LCK V12	5580	3160 (-44%)		
CD8 V12	6711	2980 (-56%)		
K-Ras V12	5411	3860 (-30%)		
N-Ras V12	4850	2560 (-48%)		
v-Src	6005	1730 (-72%)		
v-Sis	1280	650 (-49%)		
Erb-2	2400	760 (-69%)		

Supplementary Table 1. Active Ras at the Golgi Complex prevents cellular transformation. 30% confluent NIH3T3 were plated and transfected using Lipofectamine withlow amounts (0.5 ug) of the indicated plasmids and grown for 3 weeks inDMEM supplemented with 10% calf serum. The media was changed every 3days and transformation of cells and the formation of foci was monitored using microscopy and visual inspection. Result show mean of three independent experiments.

**Purpose**	**Oligonucleotide sequence**
create pME-HRAS-V12	**S**GGGGACAAGTTTGTACAAAAAAGCAGGCTCCGAATTCTA TTTGCAGCTCATGCAGCCAGG<br>**AS**GGGGACCACTTTGTACAAGAAAGCTGGGTGTTACATGA GCCAGGATCCCTCTCATCGG
create pME-KDELr-HRAS-V12	**S**GGGGACAAGTTTGTACAAAAAAGCAGGCTCCAAGCTTAT GAATCTCTTCCGATTC<br>**AS**GGGGACCACTTTGTACAAGAAAGCTGGGTCCGGATCCT GCCGGCAAACTCAA
create pME-Lck-HRAS-V12	**S**GGGGACAAGTTTGTACAAAAAAGCAGGCTAGCTTATGGG CTGTGGCTGCAGCTCA<br>**AS**GGGGACCACTTTGTACAAGAAAGCTGGGTGATCCGTTTT CCATCCAGTCATCTTCC
create pME-CD8-HRAS-V12	**S**GGGGACAAGTTTGTACAAAAAAGCAGGCTCAAGCTTATG AGACCCAGACTGTGGCTGCTG<br>**AS**GGGGACCACTTTGTACAAGAAAGCTGGGTTCACTTGTA GAATCGCTTCATGAATCTCAG
create pME-NRAS-D12	**S**GGGGACAAGTTTGTACAAAAAAGCAGGCTGGATGTACGA CGTTCCTGATTAC<br>**AS**GGGGACCACTTTGTACAAGAAAGCTGGGTGTTACATCA CCACACATGGCAATCC
create pME-BRAF-E600	**S**GGGGACAAGTTTGTACAAAAAAGCAGGCTAGGGCGAATT CCAGCAC<br>**AS**GGGGACCACTTTGTACAAGAAAGCTGGGTTCAGTGGAC AGGAAAC

qRT-PCR to zebrafish *ptprk*	**S** ACCAGAACCAAGTGTGCAGAG<br>**AS** ATGGTGACGTTGAAGGTGTG
zebrafish *ptprk* sequencing	**S** CCGTCAACAACAAACTCTG<br>**S** CTGAAGATGATCCTCACCAAC<br>**S**GACTACAACATCTACTTCCAG<br>**S**AACTCAAGGTCCTGTCCAC<br>**S** CCGAAATCACGACAAGAAC
ptprk zebrafish mutant genotyping	**S** CAGATTGTGGTGAAGGAGATC<br>**AS** CACACTGACCTTCTCCACTC
zebrafish *ptprk* cloning	**S** GACAGACAATTGGCCACCATGGATATCATCATTTTGA GC<br>**AS** GACAGAACTAGTTTATGAGGTCTCGATGAACTCCAG
Amplify DNA template to synthesise zebrafish *ptprk* guide	**S** TAATACGACTCACTATA*GGAGCCGCTGCCGTTT*ACTG GTTTTAGAGC<br>**AS** AAAAGCACCGACTCGGTGCCACTTTTTCAAG<br>T7 promoter. *target sequence* *scaffold sequence*

Supplementary Table 2 . Primer oligonucleotide sequences S – sense; AS – antisense; enzyme sites where applicable underlined.
